# How to Improve Fine Motor Skill Learning in Dentistry

**DOI:** 10.1155/2021/6674213

**Published:** 2021-02-08

**Authors:** Mohamed El-Kishawi, Khaled Khalaf, Tracey Winning

**Affiliations:** ^1^College of Dental Medicine, University of Sharjah, Sharjah, UAE; ^2^School of Dentistry, The University of Adelaide, Adelaide, SA, Australia

## Abstract

**Introduction:**

Restorative dental treatment is a complex task involving various procedures which require the development and integration of both theoretical knowledge and fine motor skills. It aims to provide the theoretical background and role of key factors in learning these skills.

**Materials and Methods:**

The following electronic databases were searched to identify relevant articles to our topic: PubMed, Medline, Google Scholar, and Scopus. Generic keywords, that is, factors, fine, performance, and dentistry, and MeSH terms, that is, “learning,” “instruction,” “patient simulation,” “motor skills,” “perception,” “tactile,” “neurophysiology,” and “working memory” were used to conduct our comprehensive search. *Results and Conclusions.* Several techniques are used in performing different restorative procedures in dentistry, that is, root canal preparation, root planning, and minor oral surgery procedures. Mastering these techniques requires a good understanding of the underpinning cognitive, sensory, and neuromuscular processes. Factors including the amount and timing of instructions provided, cognitive abilities, and practice schedule of learning trials may have significant implications on the design of fine motor skill learning exercises.

## 1. Introduction

Dental students learn the required skills as depicted in their BDS program in two stages, namely, preclinical simulated clinical activities followed later by carrying out clinical activities on patients. Due to the high cost and demand on the resources to run these simulated activities, it is important to optimize the methods of learning and make sure that these methods are informed by evidence [1–3].

There has been a limited number of previous studies reporting the rational and design of learning activities in both the preclinical and clinical dental practice [1,4–23]. Furthermore, there is only limited research investigating the factors that affect the performance of fine motor skills in dentistry. Thus, further investigations are indicated regarding the design of approaches for supporting students' learning of fine motor skills required for dental procedures that are informed by contemporary learning theories. The purpose of this review is to explore the available body of knowledge related to learning fine motor skills in dentistry. It also aims to provide the theoretical background and role of key factors in learning these skills.

## 2. Methods

The search was conducted independently by two authors who met, discussed the outcome of their searches, and agreed on the studies which were deemed to be included in this review. The following electronic databases were used: PubMed, Medline, Google Scholar, and Scopus until July 2020. Generic keywords, that is, factors, fine, performance, and dentistry, and MeSH terms, that is, “learning,” “instruction,” “patient simulation,” “motor skills,” “perception,” “tactile,” “neurophysiology,” and “working memory” were used to conduct our comprehensive search.

Inclusion criteria were as follows: all types of studies investigating factors affecting fine motor skills of dental students enrolled in either undergraduate or postgraduate programs. All included studies should have investigated one or more of the following factors: cognitive, sensory, and neuromuscular abilities of the learner, instructions, time of the clinical training, and variation in practice.

## 3. Learning Dental Skills

It is well established that the learning theory and techniques in dentistry can be challenging for undergraduate dental students [14,24–26]. Students are required to gain essential knowledge and develop related practical skills in a relatively short period of time. Specifically, they need to integrate their theoretical knowledge and motor skills and show improvement in performance to achieve the competencies required to provide patient care.

For example, learning the endodontic skills often begins with simulated activities of the different stages of root canal treatment on extracted human teeth. Cleaning and shaping of the root canal space is an essential step [27–29], aimed at eliminating or minimising the number of microorganisms causing infection in the root canal system. When using extracted human teeth, variable external and internal anatomies, as well as the condition of the root, make the instrumentation of root canal systems a challenging and sometimes discouraging task. Therefore, a recent recommendation for the simulation stage of learning endodontic procedures is to use simulated plastic models of canals and teeth prior to the use of extracted human teeth [30].

Using simulated root canals permits standardisation of the root canal hardness, length, width (diameter), location, and degree of canal curvature. This standardisation allows reproducibility of outcomes [31]. Consistent with the recommendations of learning using simulated root canals, simulated plastic blocks and teeth have been found to be a valuable adjunct for learning how to determine root canal working lengths [32] and how to carry out preparation techniques [33,34]. Studies have used simulated root canals (e.g., resin blocks, plastic teeth, and artificial dentine) to investigate and compare the shaping ability of instruments, compare different root canal instrumentation techniques, and identify possible procedural errors during root canal preparation [31,35–37]. However, how realistically simulated canals in resin teeth or blocks mimic canals in natural teeth is unclear. For example, differences in properties between resin and dentine may be an issue. Microhardness of root canal dentine has been reported to be 35–40 kg/mm^2^ compared with 20–22 kg/mm^2^ for clear resin endodontic blocks and 25–26 kg/mm^2^ for artificial resin teeth [31,38,39]. Moreover, it has been reported that the size of shavings resulting from resin and dentine is different, leading to more canal blockages in resin simulated root canals [38]. Despite these concerns, simulated root canal models have been reported to be a suitable alternative for natural teeth in learning root canal preparation procedures [34,40].

Complex dental procedures such as root canal treatment and root planning add a further complication for novice students. They do not have visual cues to support their linking and transferring their theoretical knowledge of tooth morphology and preparation techniques to appropriately complete the dental task. For example, recommendations from Australian Society of Endodontology and guidelines from the European Society of Endodontology support the use of visual demonstrations (observation) of simulated root canal procedures and techniques during learning [30,41].

## 4. Quality of Dental Procedures

Assessment of the quality of dental procedures can be achieved clinically and radiographically [42]. Clinically, accuracy of some dental tasks (e.g., canal preparation) can be determined through a tactile digital sense by inserting the hand instrument in the root canal, checking that the instrument can smoothly reach to the full working length of the canal [43]. This can then be confirmed radiographically by measuring the distance from the tip of the instrument to 0.5 to 1 mm short of the radiographic end of the root canal [29].

These procedures and techniques using hands require tactile feedback (e.g., feeling the canal walls with the hand instruments), involving somatosensory input through the fingertips, neuromuscular mediation processes, and use of correct decisions regarding the forces applied on the hand instrument during these procedures (i.e., cognitive processes).

## 5. Neurophysiology of Fine Motor Skill Learning and Control

Motor skill learning involves a continuous interaction between cognitive, sensory, and neuromuscular processes [44]. Specifically, learning a fine motor skill, as in endodontics, requires control and integration of posture, motion, and muscle stimulation that, in turn, allows the performer to execute a variety of motor behaviours that are controlled by a range of task requirements [45].

To understand how motor skills are acquired and retained, it is important to identify the mechanisms of motor activity in the human brain. Many attempts have been made to understand and determine the specialized areas of the brain responsible for motor activity [46,47]. The use of advanced techniques to monitor brain activity (including functional magnetic resonance imaging (fMRI), repetitive transcranial magnetic simulation, and electroencephalography (EEG) power spectral analysis) have allowed scientists to observe brain activity during motor tasks [48,49].

**Figure 1:**
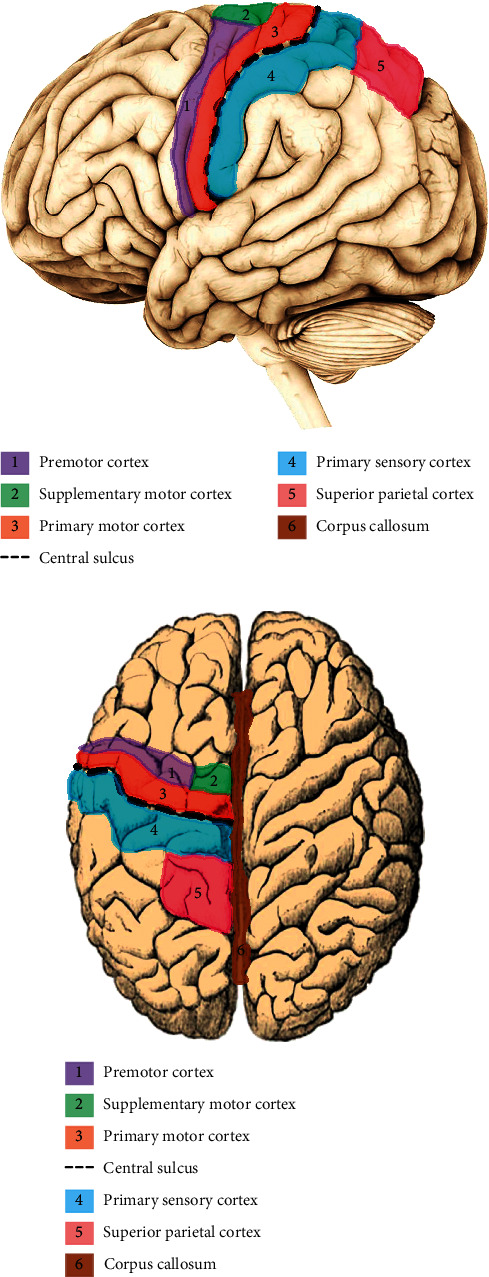
(a, b) The cortex seen from two different aspects showing parts of the brain involved in fine motor movement (modified from 65).

Using these techniques, these authors have identified six areas of the brain that play major roles in fine motor movement, including the primary motor cortex, premotor cortex, presupplementary cortex and basal ganglia, supplementary cortex, posterior parietal cortex, and cerebellum (Figure 1). Specifically, the primary motor cortex is involved in force initiation, task-specific muscle movement, and automated nature of learned movements. The premotor cortex is essential in the initial phase of learning psychomotor skills. It has an important role in movement planning, limb movement execution, and recognition. It has been demonstrated that, during nonautomated voluntary movements, the basal ganglia are active, and the presupplementary motor area is functional when learning new sequences [48].

The supplementary motor area facilitates self-initiation of movements, sequencing of previously memorised movements, two-handed coordination, and planning of complex movements. Visual response of limb movements is achieved through the posterior parietal cortex and premotor cortex. The coordination, timing, and accuracy of movements are controlled by the cerebellum, which plays a further critical role in motor learning. In particular, mediation of the voluntary movement program is achieved by the lateral cerebellum; however, motor commands are reorganised during performance by the intermediate part of the cerebellum [50]. While these areas have individual roles, as noted, they function together in harmony to enable completion of a motor task.

## 6. Sensory Input during Fine Motor Skill Learning

Brain activity related to learning fine motor skills is triggered mainly by visual and tactile sensory input systems [51]. Root canal preparation, using hand instruments principally, involves tactile (i.e., digit-sense) sensory input rather than visual input as in routine cavity preparation tasks. This involves the ability to recognise and distinguish the form of an object through exploration (touch) using indications about the texture, size, and spatial properties and temperature of the object [52,53]. It includes a mixture of somatosensory perceptions of patterns on the skin surface (e.g., edges, curvature, and texture) and proprioception of hand position and conformation [54]. In human physiology, touch and proprioception are considered as senses in the somatic sensory system and are classified into “deep sensation” related to subdermal muscles, tendons, and joints and “cutaneous sensation” that involves receptors on the surface of the skin [55]. Deep sensation occurs due to activation of receptors existing in joints and muscles and provides motion-related information like position sensation, sensation of speed, and haptic sensation [56].

Other information generated during gross and fine motor activities includes kinesthesia (i.e., movement sensitivity), which relates to the specialized sensor groups that can provide details on the length of muscles, angles of joints, degree of muscle tension, and rates of change in these values [57]. Kinesthetic information is extracted mainly from the body's physical activity, which might be autogenerated or externally reinforced. As reviewed in Gallagher and O'Sullivan [57], kinesthesia is associated with essential abilities such as walking, stretching, and grasping. It is also essential for fine motor activities (e.g., motions generated during root canal hand instrumentation, root planning, and minor oral surgery procedures), which involve specific control over the movement and position of body parts. Kinesthetic receptors are located in muscles, tendons, and linings of joints. These receptors react to mechanical force (e.g., rotations and pressure forces during hand instrumentation), which might be produced by stretching a muscle, pulling a tendon, or bending a joint [57].

Tactile sensory information plays an important role in improving motor skill control and performance [53]. Researchers have found that tactile feedback from fingertips is essential for defining characteristics of movement, including movement accuracy (i.e., grip precision and movement sequence) [58,59], movement consistency, ongoing movement force adjustment, and aiding proprioceptors to estimate the beginning and end of a movement [51]. For example, it is expected that tactile feedback is critical to improve hand-instrument grasp, judge the amount of pressure and force to be applied on hand instruments during instrument rotations, and estimate the start and end points of each of the rotations. Moreover, this will improve special awareness through direct and indirect visualization during the fine motor skill procedure, such as cavity preparation, root canal preparation, root planning, and minor oral surgery procedures.

## 7. Factors Affecting Motor Skill Learning

Dental clinical practice is complex and the design and application of an appropriate motor learning strategy are often multifactorial [60]. Therefore, it is important to identify factors that can influence the choice of motor learning strategy and how to translate clinical theory into practical actions. Many factors have been found to facilitate motor skill learning. These factors include instructions, type and timing of feedback, type of task, stage of learning, abilities related to the learner (e.g., working memory), repetition and variation of practice, and manual guidance [61,62]. In this review, the focus will be on the role of instructions, abilities related to the learner, and variation in practice in fine motor skill learning during root canal preparation. These factors have specific implications for the designs of fine motor skill learning exercises in endodontics.

### 7.1. Instructions

There is strong evidence that supports the value of verbal instructions in shaping motor skill learning [61,63]. To optimize learning outcomes, it is suggested that the quantity of verbal instructions is minimal and should not exceed the learner's attentional capacity. Instructions during motor skill learning often include descriptions of the movements of a particular part (s) of the body (e.g., hand or fingers) in relation to other body parts in space and time [64]. This type of instruction, focusing on specific body movements, is referred to as having an “internal focus.” In contrast, instructions that direct a learner's attention to the effect of the movement are referred to as having an “external focus” [65]. Studies on attentional focus effects have shown that minor alterations in the wording of instructions can have a major effect on learning and performance [64]. Applying this in root canal preparation task, it seems that providing instructions characterised by an external focus of attention (e.g., caries removal and the final shape of cavity preparation) has more learning advantages in contrast with an internal focus of attention (e.g., angulation of the bur, movement, or grasp on a handpiece).

The advantage of an external focus can be explained by the utilisation of unconscious and automated processing of information related to the task [64]. Use of this automated information can accelerate the learning process and shorten the initial stage of learning. In contrast, using an internal focus, learners tend to get confused due to the incompatibility of the information provided with their movement planning and desired outcome, resulting in conscious concentration on the control of movement [65]. However, Poolton, Maxwell, Masters, and Raab [66] examined the effect of attentional focus on learning and performance of a complex motor task and suggested that deterioration of performance in the internal focus of attention group was related to generating greater attentional demands on working memory compared with the instruction based on an external focus of attention.

### 7.2. Abilities of the Learner

Memory plays an important role during learning [67]. The structure of memory consists of two memory function systems, namely, working memory (i.e., short-term memory) and long-term memory [68]. During motor skill learning, visual, auditory, proprioceptive, and tactile sensory forms of information are temporarily stored in working memory. These types of information are made available to be used for assessment of outcomes and performance [69]. When processing novel information, the duration and capacity of working memory are limited. It has been shown that movement information stored in working memory tends to be lost (i.e., forgotten) after about 20–30 seconds [67]. The scope of short-term working memory is also limited. This limitation can affect the amount of information that can be received, processed, and stored in working memory [69]. Based on Miller's [70] suggestion, the capacity of working memory is about seven items, plus or minus two items. For example, in relation to motor skill learning, working memory can hold 7 ± 2 procedural instructions or rules related to movements and movement sequences [69].

The second component of the memory system is long-term memory. Long-term memory functions as a permanent store for information. Procedural memory is the part of long-term memory which stores and retrieves motor skill information [69]. These skills are difficult to be described verbally but are rather expressed by means of performance [71]. Procedural memory is essential for performance of a motor skill as a learned procedure is evaluated based on the produced actions rather than verbalisation of the actions. [69] As reviewed by Magill and Anderson [69], both working and long-term memory systems interact with each other, and distinctions in the functions of each system depend on the level of performance and stage of learning during motor skill acquisition and performance.

During motor skill acquisition, the learner progresses through three stages of development: the cognitive (declarative) stage; associative (knowledge compilation) stage; autonomous (procedural) stage [72]. In the declarative stage, execution of a motor skill relies on an unintegrated collection of rules stored in working memory that are used to control and guide performance [72]. This process depends on working memory such that working memory capacity is reduced relative to rules in use which leads to a reduction in the capacity to interpret and process other information related to performance of the task. During the associative and procedural stages, further prolonged application of these rules occurs until the motor skill is acquired, resulting in automation of the motor skill [73,74]. In relation to dental procedures, the cognitive stage is represented by a student's reliance mainly on verbal instructions provided to complete the dental tasks. Following initial practice, students will interpret these instructions to improve their performance. During the procedural stage, students would be familiar with the instructions and rules, resulting in performing the dental procedure without reliance on these instructions.

### 7.3. Variation in Practice

**Figure 2:**
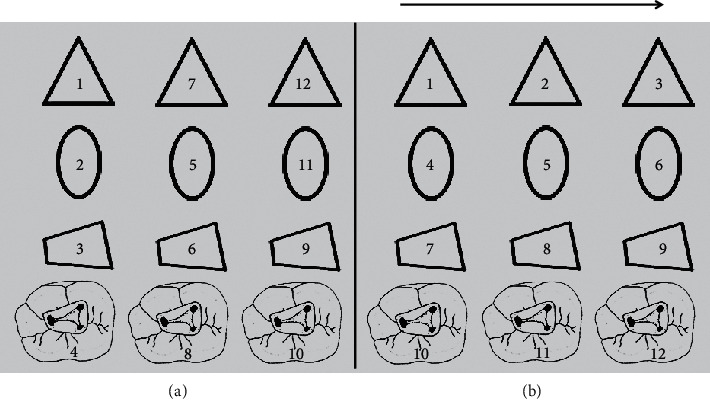
Simulated access cavity preparation on plastic training blocks. (a) Random practice order. (b) Blocked practice order.

Research in the motor learning domain has highlighted the importance of practice variables on motor learning (e.g., practice schedule) [61]. Studies comparing a blocked (i.e., repetitive) practice schedule (i.e., AAA, BBB, and CCC) to a random (i.e., unpredictable) practice schedule (i.e., ABC, BCA, and CAB) (Figure 2) during learning trials have found that blocked practice results in superior performance to random practice [75]. In contrast, this study also showed that random practice results in superior retention of performance compared with blocked practice. Random practice is suggested to create an episodic retention loss during practice and subsequent reconstruction, which disadvantages performance relative to blocked practice but is beneficial to retention of learning following practice. However, these findings were only applicable to relatively simple tasks (e.g., key-press sequence) but not complex tasks [76]. When performing a complex task (e.g., root canal hand instrumentation), random practice would result in increasing attentional demands on working memory resources due to hypothesis testing to correct unsuccessful attempts. This overload on working memory disrupts the automated execution of some of the motor skill components, resulting in the loss of flexibility of the movement, and thereby potentially causing deterioration of performance. Therefore, it is suggested that blocked practice (i.e., practicing the entire skill) would be more beneficial when the task is complex [61,76].

## 8. Implications on Fine Motor Skill Learning in Dentistry

Based on the outcomes of our study, the following points should be considered during clinical training.

  Simulated root canal models have been reported to be a suitable alternative for natural teeth in learning restorative procedures.
  Tactile sensory information feedback is critical to improve hand-instrument grasp, judge the amount of pressure, direction, and force to be applied on hand instruments during instrument rotations, and estimate the start and end points of each of the movements.
  To optimize learning outcomes, it is suggested that the quantity of verbal instructions is minimal and should not exceed seven items, plus or minus two items.
   Instructions that direct a learner's attention to the effect of the movement (external focus) can accelerate the learning process and have more learning advantages in contrast with an internal focus of attention (e.g., angulation of the bur, movement, or grasp on a handpiece).
   Given the complex nature of dental procedures, blocked practice schedule (i.e., AAA, BBB, and CCC) results in superior performance to random practice schedule (i.e., ABC, BCA, and CAB).

## 9. Conclusion

Several techniques are used in performing different restorative procedures in dentistry, for example, root canal preparation, root planning, and minor oral surgery procedures. Mastering these techniques requires a good understanding of the underpinning cognitive, sensory, and neuromuscular processes. Simulated models have been reported to be a suitable alternative for natural teeth in fine motor skills learning; however, further research is needed to improve the quality and design of these models. Factors including the amount and timing of instructions provided, cognitive abilities, and practice schedule of learning trials may have significant implications on the design of fine motor skill learning exercises.

## Data Availability

The data used to support the findings of this study are included within the article.

## References

[1] Tedesco L. (1995). Issues in dental curriculum development and change. *Journal of Dental Education*.

[2] Glickman G., Gluskin A., Johnson W., Lin J. (2005). The crisis in endodontic education: current perspectives and strategies for change. *Journal of Endodontics*.

[3] McNally M., Dunning D., Lange B., Gound T. (2002). A survey of endodontic residents’ attitudes about a career in dental education. *Journal of Endodontics*.

[4] Feil P., Guenzel P., Knight G., Geistfeld R. (1994). Designing preclinical instruction for psychomotor skills (I)-theoretical foundations of motor skill performance and their applications to dental education. *Journal of Dental Education*.

[5] Knight G., Guenzel P., Feil P. (1994). Designing preclinical instruction for psychomotor skills (II)-instructional engineering: task analysis. *Journal of Dental Education*.

[6] Knight G., Guenzel P., Feil P. (1997). Using questions to facilitate motor skill acquisition. *Journal of Dental Education*.

[7] Boyd M., Wood W., Conry R. (1980). Prediction of preclinical operative dentistry performance in two instructional methods. *Journal of Dental Education*.

[8] Quinn F., Keogh P., McDonald A., Hussey D. (2003). A study comparing the effectiveness of conventional training and virtual reality simulation in the skills acquisition of junior dental students. *European Journal of Dental Education*.

[9] Wierinck E. R., Puttemans V., Swinnen S. P., van Steenberghe D. (2007). Expert performance on a virtual reality simulation system. *Journal of Dental Education*.

[10] Winning T., Malhotra N., Masters R. S. W. (2018). Investigating an errorless learning approach for developing dental operative technique skills: a pilot study. *European Journal of Dental Education*.

[11] Koedijker J. M., Poolton J. M., Maxwell J. P., Oudejans R. R. D., Beek P. J., Masters R. S. W. (2011). Attention and time constraints in perceptual-motor learning and performance: instruction, analogy, and skill level. *Consciousness and Cognition*.

[12] Abou-Rass M. (1974). Effects of varying sequence and amount of training on learning and performance in preclinical endodontics. II. Study on amount of training. *Journal of Dental Education*.

[13] Abou-Rass M. (1974). Effects of varying sequence and amount of training on learning and performance in preclinical endodontics. Part I: design, experimental procedures, and sequencing. *Journal of Dental Education*.

[14] Friedlander L., Anderson V. (2011). A new predoctoral endodontic module: evaluating learning and effectiveness. *Journal of Dental Education*.

[15] Sonntag D., Bärwald R., Hülsmann M., Stachniss V. (2008). Pre-clinical endodontics: a survey amongst German dental schools. *International Endodontic Journal*.

[16] Qualtrough A. J. E., Whitworth J. M., Dummer P. M. H. (1999). Preclinical endodontology: an international comparison. *International Endodontic Journal*.

[17] Petersson K., Olsson H., Söderström C., Fouilloux I., Jegat N., Lévy G. (2002). Undergraduate education in endodontology at two European dental schools. *European Journal of Dental Education*.

[18] Qualtrough A. J. E., Dummer P. M. H. (1997). Undergraduate endodontic teaching in the United Kingdom: an update. *International Endodontic Journal*.

[19] Chandler N., Bloxham G. (1990). The influence of two handle designs and gloves on the performance of a simulated endodontic task. *Journal of Endodontics*.

[20] Chandler N. P., Robyn Shaw J., Treble S. J. (1996). Effect of endodontic instrument handle diameter on operator performance. *Journal of Endodontics*.

[21] Treble S. J., Chandler N. P., Shaw J. R. (1993). Tactile sensitivity of three endodontic instrument handles. *Dental Traumatology*.

[22] Chandler N. P., Pitt Ford T. R., Monteith B. D. (2004). Pulp size in molars: underestimation on radiographs. *Journal of Oral Rehabilitation*.

[23] Min L., Yun-hui L., Qiang H. An optimized haptic interaction model based on support vector regression for evaluation of endodontic shaping skill, Robotics and Biomimetics.

[24] Burrell W., Rasmussen R. (1977). An evaluation system for preclinical and clinical endodontics. *Journal of Dental Education*.

[25] Seijo M. O. S., Ferreira E. F., Ribeiro Sobrinho A. P., Paiva S. M., Martins R. C. (2013). Learning experience in endodontics: brazilian students’ perceptions. *Journal of Dental Education*.

[26] Murray C. M., Chandler N. P. (2014). Undergraduate endodontic teaching in New Zealand: students’ experience, perceptions and self-confidence levels. *Australian Endodontic Journal*.

[27] Peters O. (2004). Current challenges and concepts in the preparation of root canal systems: a review. *Journal of Endodontics*.

[28] Schilder H. (1974). Cleaning and shaping the root canal. *Dental Clinics of North America*.

[29] Peters O., Peters C., Hargreaves K., Cohen S., Berman L. (2010). Cleaning and shaping of the root canal system. *Cohen’s Pathways of the Pulp*.

[30] Australian Society of Endodontology (2007). *Revised Guidelines for Educational Requirements for Undergraduate Training in Endodontics*.

[31] Lim K. C., Webber J. (1985). The validity of simulated root canals for the investigation of the prepared root canal shape. *International Endodontic Journal*.

[32] Tchorz J. P., Ganter P. A., Woelber J. P., Stampf S., Hellwig E., Altenburger M. J. (2014). Evaluation of an improved endodontic teaching model: do preclinical exercises have an influence on the technical quality of root canal treatments?. *International Endodontic Journal*.

[33] LaTurno S. A. L., Corcoran J. F., Ellison R. L. (1984). An evaluation of a teaching aid in endodontics. *Journal of Endodontics*.

[34] Nassri M. R. G., Carlik J., Silva C. R. N. D., Okagawa R. E., Lin S. (2008). Critical analysis of artificial teeth for endodontic teaching. *Journal of Applied Oral Science*.

[35] Weine F. S., Kelly R. F., Lio P. J. (1975). The effect of preparation procedures on original canal shape and on apical foramen shape. *Journal of Endodontics*.

[36] Hülsmann M., Peters O. A., Dummer P. M. H. (2005). Mechanical preparation of root canals: shaping goals, techniques and means. *Endodontic Topics*.

[37] Alodeh M. H. A., Doller R., Dummer P. M. H. (1989). Shaping of simulated root canals in resin blocks using the step-back technique with K-files manupulated in a simple in/out filing motion. *International Endodontic Journal*.

[38] Weine F., Kelly R., Bray K. (1976). Effect of preparation with endodontic handpieces on original canal shape. *Journal of Endodontics*.

[39] Nissin Dental (2013). *Microhardness of Nissin^TM^ Endodontic Training Resin Blocks and Teeth*.

[40] Tchorz J. P., Brandl M., Ganter P. A. (2014). Pre-clinical endodontic training with artificial instead of extracted human teeth: does the type of exercise have an influence on clinical endodontic outcomes?. *International Endodontic Journal*.

[41] De Moor R., Hülsmann M., Kirkevang L.-L., Tanalp J., Whitworth J. (2013). Undergraduate curriculum guidelines for endodontology. *International Endodontic Journal*.

[42] Ingle J., Bakland L., Baumgartner J. (2008). *Ingle’s Endodontics*.

[43] Peters O., Koka R., Ingle J., Bakland L., Baumgartner J. (2008). Preparation of coronal and radicular spaces. *Ingle’s Endodontics*.

[44] Mulder T., Hochstenbach J., Greenwood R., McMillan T., Barnes M., Ward C. (2002). Motor control and learning: implications for neurological rehabilitation. *Handbook of Neurological Rehabilitation*.

[45] Newell K. M. (1991). Motor skill acquisition. *Annual Review of Psychology*.

[46] Gerloff C., Corwell B., Chen R., Hallett M., Cohen L. (1998). The role of the human motor cortex in the control of complex and simple finger movement sequences. *Brain*.

[47] Watson A. H. D. (2006). What can studying musicians tell us about motor control of the hand?. *Journal of Anatomy*.

[48] Toni I., Krams M., Turner R., Passingham R. E. (1998). The time course of changes during motor sequence learning: a whole-brain fMRI study. *Neuroimage*.

[49] Zhu F. F., Poolton J. M., Wilson M. R., Maxwell J. P., Masters R. S. W. (2011). Neural co-activation as a yardstick of implicit motor learning and the propensity for conscious control of movement. *Biological Psychology*.

[50] Seitz R. J., Roland P. E. (1992). Learning of sequential finger movements in man: a combined kinematic and positron emission tomography (PET) study. *European Journal of Neuroscience*.

[51] Kemble J. V., Anderson D. (1975). PH changes on the surface of burns. *British Journal of Plastic Surgery*.

[52] Boehm A. (1941). Stereognosis and tactile “auto-sensations“1. *British Journal of Psychology. General Section*.

[53] Lederman S. J., Klatzky R. L. (1993). Extracting object properties through haptic exploration. *Acta Psychologica*.

[54] Streri A., Spelke E. S. (1988). Haptic perception of objects in infancy. *Cognitive Psychology*.

[55] Hayashi K., Takahata M. (2005). Objective evaluation of tactile sensation for tactile communication. *NTT DoCoMo Technical Journal*.

[56] Lederman S. J., Klatzky R. L. (2009). Haptic perception: a tutorial. *Attention, Perception & Psychophysics*.

[57] Gallagher A. G., O’Sullivan G. C. (2012). *Human Factors in Acquiring Medical Skills; Learning and Skill Acquisition in Surgery, Fundamentals of Surgical Simulation Principles and Practices*.

[58] Goebl W., Palmer C. (2008). Tactile feedback and timing accuracy in piano performance. *Experimental Brain Research*.

[59] Fisher R. J., Galea M. P., Brown P., Lemon R. N. (2002). Digital nerve anaesthesia decreases EMG-EMG coherence in a human precision grip task. *Experimental Brain Research*.

[60] Elani H. W., Allison P. J., Kumar R. A., Mancini L., Lambrou A., Bedos C. (2014). A systematic review of stress in dental students. *Journal of Dental Education*.

[61] Kleynen M., Braun S., Rasquin S. (2015). Multidisciplinary views on applying explicit and implicit motor learning in practice: an international survey. *PLoS One*.

[62] Kleynen M., Braun S., Bleijlevens M. (2014). Using a Delphi technique to seek consensus regarding definitions, descriptions and classification of terms related to implicit and explicit forms of motor learning. *PLoS One*.

[63] Stamm O., Latscha U., Janecek P., Campana A. (1976). Development of a special electrode for continuous subcutaneous pH measurement in the infant. *American Journal of Obstetrics and gynecology*.

[64] Campana G., Shea C., Lewthwaite R. (2010). Motor skill learning and performance: a review of influential factors. *Medical Education*.

[65] Wulf G., Prinz W. (2001). Directing attention to movement effects enhances learning: a review. *Psychonomic Bulletin & Review*.

[66] Poolton J. M., Maxwell J. P., Masters R. S. W., Raab M. (2006). Benefits of an external focus of attention: common coding or conscious processing?. *Journal of Sports Sciences*.

[67] Cowan N. (2014). Working memory underpins cognitive development, learning, and education. *Educational Psychology Review*.

[68] Baddeley A. (2003). Working memory: looking back and looking forward. *Nature Reviews Neuroscience*.

[69] Magill R. A., Anderson D. L. (2013). *Memory Components, Forgetting, and Strategies, Motor Learning and Control: Concepts and Cpplications*.

[70] Miller G. (1956). The magical number seven, plus or minus two: some limits on our capacity for processing information. *Psychological Review*.

[71] Ten Berge T., Van Hezewijk R. (1999). Procedural and declarative knowledge. *Theory Psychol*.

[72] Anderson J. (1982). Acquisition of cognitive skill. *Psychological Review*.

[73] Fitts P., Posner M. (1967). *Human Performance, Belmont, Calif*.

[74] Maxwell J., Masters R., Eves F. (2003). The role of working memory in motor learning and performance. *Consciousness and Cognition*.

[75] Li Y., Wright D. (2000). An assessment of the attention demands during random- and blocked-practice schedules. *The Quarterly Journal of Experimental Psychology Section A*.

[76] Akizuki K., Ohashi Y. (2013). Changes in practice schedule and functional task difficulty: a study using the probe reaction time technique. *Journal of Physical Therapy Science*.

